# Genomic Evaluation of the Genetic Structure and Analysis of Selective Evolutionary Signatures of Xupu Goose

**DOI:** 10.3390/biology15060479

**Published:** 2026-03-17

**Authors:** Kairui Zhu, Zhenkang Ai, Yuchun Cai, Yonghao Li, Yuhang Cheng, Yang Zhang, Wenming Zhao, Guohong Chen

**Affiliations:** 1 College of Animal Science and Technology, Yangzhou University, Yangzhou 225009, China; 2 Key Laboratory for Evaluation and Utilization of Livestock and Poultry Resources (Poultry), Ministry of Agriculture and Rural Affairs, Beijing 100125, China

**Keywords:** Xupu goose, whole-genome resequencing, population genetic structure, selective sweeps, IHS selection signals, genetic diversity

## Abstract

As an elite indigenous poultry breed under national protection in China, the Xupu goose is renowned for its large body size, superior fatty liver production, premium meat quality, and high tolerance to roughage. To elucidate its genomic architecture, genetic diversity, and evolutionary selection signatures, we conducted whole-genome resequencing on 15 purposively selected, unrelated male Xupu geese. An average of 6.79 Gb of high-quality sequence data was generated per individual, yielding approximately 4.27 million single-nucleotide polymorphisms (SNPs) with a transition/transversion (Ti/Tv) ratio of 2.49. Population genomic analyses revealed that while the population retains a moderate genetic reservoir (*H_E_* = 0.298), it exhibits a distinct heterozygote deficit (*H_O_* = 0.217) and a moderate genomic inbreeding coefficient *F_ROH_* = 0.204). This structural pattern underscores the genetic impact of historical ex situ closed-flock conservation and the consequent formation of cryptic family lineages. Furthermore, genome-wide integrated haplotype score (iHS) scans detected distinct regions under recent positive selection. Functional annotation of these regions highlighted candidate genes tightly associated with the breed’s hallmark traits, specifically lipid metabolism and hepatic fat deposition (*ACSS2*, *ACSS3*, *PECR*), alongside muscle development (*CMYA5*, *MTPN*, *LEPR*). Conclusively, this study delineates a comprehensive genomic landscape of the Xupu goose, providing a robust foundational resource for future germplasm conservation, molecular marker development, and precision breeding programs.

## Simple Summary

The Xupu goose is an economically valuable indigenous breed in China, celebrated for its large body size and exceptional fatty liver capacity. However, as an “at-risk” population, it requires urgent conservation efforts. In this study, we conducted whole-genome sequencing on 15 Xupu geese to evaluate their genetic diversity and uncover the genomic basis of their unique traits. We found that while the breed retains a moderate genetic reservoir, it carries a legacy of historical inbreeding driven by past population bottlenecks. Importantly, our results indicate that current conservation practices have successfully prevented severe recent inbreeding, despite the natural emergence of distinct family lineages. Furthermore, we identified key candidate genes strongly linked to lipid metabolism and muscle development. These findings provide crucial scientific guidance for optimizing future mating strategies and lay the genomic groundwork for the precision breeding of this vital waterfowl.

## 1. Introduction

China’s vast territory and diverse ecological types have nurtured abundant indigenous goose genetic resources with distinct characteristics [1,2]. These indigenous breeds are widely distributed across China and have developed strong adaptability to complex local environments through long-term natural domestication and artificial selection [3]. Among them, the Xupu goose, originating from the hilly regions of Xupu County in Hunan Province and recognized as one of the “Three Famous Geese of China”, is renowned for its large body size, exceptional fatty liver performance, high-quality down, and tolerance to coarse fodder. Studies have shown that its meat is rich in unsaturated fatty acids, and the average weight of its fatty liver can exceed 606 g [4]. Furthermore, conservation assessments by Chen et al. classify the Xupu goose as “at-risk” among Chinese indigenous waterfowl resources [5]. In recognition of its exceptional breed characteristics and urgent conservation value, the breed has been accorded high-level national protection status. Specifically, the Xupu goose was inscribed in the National List of Livestock and Poultry Genetic Resources for Conservation in February 2014 and was subsequently designated as a National Geographical Indication Protected Product in December of that year.

Whole-genome resequencing (WGS), relying on reference genomes, utilizes high-throughput technology to accurately identify single-nucleotide polymorphisms (SNPs), insertions/deletions (Indels), and structural variations (SVs) within the genome [6,7]. These genetic variation data are instrumental in revealing the genetic diversity, selective evolutionary signals, and domestication processes of goose populations [8,9]. In studies of breed origins, Wen et al. [10] confirmed the dual-origin model of domestic geese—originating from Swan Geese (*Anser cygnoides*) and Greylag Geese (*Anser anser*)—through resequencing analysis of domestic geese and their wild ancestors, and elucidated the impact of gene introgression, such as *IGF-1*, on body size evolution. Regarding phenotypic trait dissection, Ren et al. [11] identified 26 plumage color regulatory genes, including *KITLG*, using Pool-Seq technology. Sun et al. [12] mapped 88 SNP loci significantly associated with body size through GWAS, revealing the regulatory mechanisms of genes like *THADA* in skeletal development. For reproductive traits, Zhao et al. [13] screened 107 egg-production-related candidate genes and found them significantly enriched in signaling pathways such as PI3K-Akt. In summary, whole-genome data serves not only as the cornerstone for genetic resource conservation but also as a key driver for transitioning the goose industry toward molecular precision breeding.

The unique lineage of the Xupu goose is deeply rooted in its geographic and cultural history. As documented in the 1869 edition of the Xupu County Annals, the breed emerged through over a century of localized, closed-herd breeding, with residents rarely transferring populations outside the hilly regions of Hunan Province. While this unique breeding history has shaped its superior breed characteristics, it also exposes the population to the potential risk of reduced genetic diversity. However, a systematic genomic evaluation of the Xupu goose—particularly regarding its genetic diversity, inbreeding levels (ROH), and selective signatures—remains insufficient. This study aims to conduct a whole-genome resequencing analysis of Xupu geese to deeply explore their genetic variation characteristics, thereby providing systematic theoretical support for the conservation and molecular breeding of this breed.

## 2. Materials and Methods

### 2.1. Ethics Statement

To ensure the highest level of Animal Ethics and welfare, this study was conducted under the rigorous oversight of the committee at Yangzhou University. All protocols were officially authorized under Permit Number 2023004742, ensuring that every step of the research met established safety and humanitarian guidelines. Beyond institutional approval, the project maintained full Regulatory Compliance with the “Regulations on the Administration of Laboratory Animal Affairs” (Yangzhou University, 2012) and “the specific Management Standards for Experimental Practices” (Jiangsu, China, 2008) established in Jiangsu, China. By integrating these legal frameworks, the study guarantees that all animal management procedures were performed with professional integrity and in accordance with modern scientific standards.

### 2.2. Experimental Animals

In this genetic study, male Xupu geese were obtained from the National Waterfowl Gene Bank (Taizhou, China), where the conservation population is maintained under a closed management system utilizing rotational mating. To maximize breed representativeness and minimize kinship bias, experimental individuals were randomly selected from distinct, unrelated families based on comprehensive pedigree records. Venous blood samples (2 mL) were collected from the brachial vein of 15 male geese (90 days old) using sterile heparinized syringes. Blood collection was performed by licensed veterinarians. To ensure animal welfare, geese were gently restrained using a calm, manual holding technique to minimize stress. No anesthesia was required for the minimally invasive wing vein puncture, and hemostasis was immediately confirmed after sampling to prevent infection. Collected blood samples were processed for genomic DNA extraction and subsequent genetic analyses. Prior to extraction, samples were temporarily stored at −20 °C. Following extraction, the purified genomic DNA was stored at −80 °C to preserve integrity for downstream sequencing.

### 2.3. Genomic DNA Extraction and Quality Assessment

Genomic DNA was extracted from blood samples using a DNA extraction kit (DP304, Tiangen, China). The integrity and fragmentation of the extracted genomic DNA were verified using 1% agarose gel electrophoresis, and the precise concentration was determined using a NanoDrop-2000 micro-spectrophotometer (Menlo Park, CA, USA) to ensure all samples met the stringent requirements for library construction and sequencing. Only samples meeting stringent quality criteria (concentration > 200 ng/µL; OD260/280: 1.75–2.0) were selected for library construction and sequencing. Qualified DNA samples were shipped to Nanjing Jisihuiyuan Biotechnology Co., Ltd. (Nanjing, China) for resequencing library construction and sequenced on the Illumina NovaSeq 6000 platform. The *Anser cygnoides* (Swan goose) reference genome (ASM4018256v1; NCBI Accession: GCF_040182565.1) was utilized for read mapping and downstream genomic analyses. SNP calling was performed using the Genome Analysis Toolkit (GATK). After stringent filtering, the obtained SNP markers were utilized for haplotype phasing and integrated Haplotype Score (iHS) analysis [14].

### 2.4. Data Quality Control and Variant Calling

Raw data were acquired using a high-throughput sequencing platform (Illumina NovaSeq 6000, San Diego, CA, USA) and converted into raw reads. Subsequently, the sequencing reads were aligned to the reference genome using BWA. On this basis, PCR duplicates were removed using Picard tools (V2.17.0) (Mark Duplicates) to eliminate the effects of amplification bias. Subsequent variant detection was primarily implemented using the GATK (v4.1.4.1) toolkit. Specifically, variant calling was first performed to identify candidate SNPs and InDel loci, followed by Variant Quality Score Recalibration and stringent data filtering to construct the final set of high-confidence SNP loci.

### 2.5. Analysis of Population Genetic Diversity, Linkage Disequilibrium (LD), and Runs of Homozygosity (ROH)

To conduct an in-depth analysis of the genetic structure of the Xupu goose population, the --het and --freq functions in PLINK 1.9 software were employed post-filtering to accurately calculate key indices of genetic variation, including expected heterozygosity (*H_E_*) and observed heterozygosity (*H_O_*), which were subsequently used to assess the inbreeding coefficient (F) and intra-population genetic distance (D_st_) [15].

Population genetic structure is a crucial approach for investigating internal evolutionary processes and phylogenetic relationships within a species. The top 10 eigenvalues and eigenvectors were calculated using the --pca parameter in PLINK v1.9 software. Subsequently, the first two principal components (PC1 and PC2) were visualized using the ggplot2 package in R v4.4.0 to explore the clustering relationships and genetic consistency within the conserved population.

The characterization of population genetic structure is an essential approach for elucidating intraspecific evolutionary dynamics and phylogenetic relationships. To this end, Principal Component Analysis (PCA) was conducted using PLINK v1.9 (with the --pca parameter) to extract the top 10 principal components. Subsequently, the first two principal components (PC1 and PC2) were visualized using the ggplot2 package in R v4.4.0 to assess spatial clustering patterns and genetic homogeneity within the conserved population.

Linkage Disequilibrium (LD) analysis was performed using PopLDdecay (https://github.com/BGI-shenzhen/PopLDdecay, accessed on 14 February 2026) with the following specific parameters: -MaxDist 500, -Het 0.1, -Miss 0.3, and -OutPairLD 5. The chromosomal distribution of LD was visualized via LD decay plots, which characterize the relationship between the LD decay rate and physical or genetic distance [16].

To identify Runs of Homozygosity (ROH) within the Xupu goose genome, a systematic scan of the autosomes was performed using PLINK V1.9 software [17,18]. The detection process employed a sliding window approach, with stringent filtering parameters established based on the studies by Yu et al. [19] and Sun et al. [20] to ensure the reliability of the results. The detection parameters were strictly defined as follows: a sliding window of 50 SNPs was employed (--homozyg-window-snp 50), allowing for a maximum of one heterozygous genotype (--homozyg-window-het 1) and five missing genotypes (--homozyg-window-missing 5) per window, with a threshold of 0.05 (--homozyg-window-threshold 0.05). To be classified as an ROH, a segment was required to span at least 100 kb (--homozyg-kb 100), contain a minimum of 10 SNPs (--homozyg-snp 10), and maintain a minimum density of one SNP per 10 kb (--homozyg-density 10), with a maximum gap of 100 kb between adjacent markers (--homozyg-gap 100). Statistical analyses were conducted on the frequency, length, and distribution of ROH within the Xupu goose population. The genomic inbreeding coefficient based on *F_ROH_* (was calculated using the following formula [21]:

$$F_{ROH}=\frac{\sum L_{ROH}}{L_{AUTO}}$$

where $\sum L_{ROH}$ represents the total length of all ROH segments detected in each individual, and $L_{AUTO}$ is the total length of the autosomal genome covered by the genotype data.

### 2.6. Detection of Selection Signatures Using iHS

To identify genomic regions under selection (significant signatures) within the Xupu goose population, the Integrated Haplotype Score (iHS) method was employed. The standardized iHS score is typically calculated as follows [22]:

$$iHS=\frac{ln(\frac{{iHH}_{A}}{{iHH}_{D}})-E_{p}[ln(\frac{{iHH}_{A}}{{iHH}_{D}})]}{{SD}_{p}\left[ln(\frac{{iHH}_{A}}{{iHH}_{D}})\right]}$$

where ${iHH}_{A}$ and ${iHH}_{D}$ represent the integrated Extended Haplotype Homozygosity (EHH)scores for the ancestral and derived core alleles, respectively. $E_{p}[ln(\frac{{iHH}_{A}}{{iHH}_{D}})]$ and ${SD}_{p}\left[ln(\frac{{iHH}_{A}}{{iHH}_{D}})\right]$ denote the expected value (mean) and standard deviation calculated within allele frequency bin *p*. iHS scores were calculated and visualized as Manhattan plots using the *rehh* package (v3.2.1) in R. Candidate genes located within genomic regions exhibiting significant selection signatures were identified based on a threshold of |iHS| > 2 (*p* < 0.05) to filter potential false positives. Subsequently, functional enrichment analyses, including Gene Ontology (GO) terms and Kyoto Encyclopedia of Genes and Genomes (KEGG) pathways, were performed using the Metascape platform [23]. Statistically significant terms were identified using a Benjamini–Hochberg corrected *p*-value threshold of < 0.05.

## 3. Results

### 3.1. Statistics and Quality Assessment of Sample Sequencing Data

**Figure 1:**
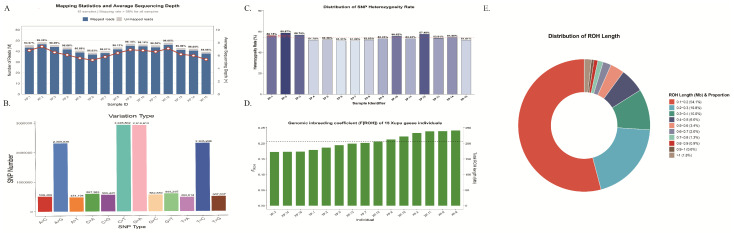
Sequencing quality, SNP variation characteristics, and genomic inbreeding patterns derived from whole-genome resequencing of Xupu geese: (**A**) Alignment statistics of sequencing reads to the reference genome and average sequencing depth. (**B**) Distribution of SNP heterozygosity rates across individuals. (**C**) Statistics of genome-wide SNP variant types. (**D**) Statistics of genome-wide SNP variant types. Genomic inbreeding coefficients *F_ROH_*) estimated based on Runs of Homozygosity (ROH). (**E**) Proportional distribution of ROH across different length categories.

In this study, whole-genome resequencing was performed on the Xupu goose, a distinctive indigenous breed, followed by a rigorous quality control pipeline for the acquired sequencing data. Data analysis results indicated that the average raw data volume per sample reached 6.87 Gb, with an average of 22,896,147 raw reads (Table 1). After stringent filtering, the average effective data volume was 6.79 Gb, with an average of 22,660,317 clean reads, yielding a high effective data utilization rate of 98.97% (Figure 1A). Regarding sequencing quality assessment, the average values for Q20 and Q30 reached 97.23% and 91.32%, respectively, with an average GC content of 43.77%. Furthermore, the GC content distribution across all samples was balanced, ranging from 43.31% to 44.52%. In summary, the sequencing data exhibited excellent accuracy and stability, fully meeting the technical requirements for genome-wide SNP variant detection and subsequent population genetics analysis, thus providing a solid data foundation.

### 3.2. Analysis of SNP Variant Detection and Annotation Results

Whole-genome SNP variant detection was performed on all samples in this study. Statistical results indicate some fluctuation in SNP counts across samples (Table 2), with an average of 4,269,107, ranging from 3,877,534 in XP.15 to 4,629,469 in XP.12. These inter-individual differences may stem from subtle variations in sequencing depth or the inherent heterogeneity of genetic backgrounds.

In terms of heterozygosity analysis, the average number of heterozygous SNPs (2,312,216) was significantly higher than that of homozygous SNPs 1,959,778, with an average Heterozygous variant proportion of approximately 54% (Figure 1A). Inter-individual analysis showed that sample XP.12 had the highest number of heterozygous loci (2,661,174), suggesting strong individual genetic polymorphism; conversely, sample XP.15 had a relatively lower number (2,008,960), suggesting this individual may have accumulated more homozygous segments.

Regarding variant types, the transition/transversion (Ti/Tv) ratio across all samples showed high consistency, ranging between 2.47 and 2.50 (mean 2.49). In molecular evolution, due to the cytosine methylation deamination effect, the biochemical probability of transitions is significantly higher than that of transversions. The ratio of 2.49 obtained in this study aligns with the typical characteristics of most vertebrate genomes, usually between 2.0 and 2.5, indicating not only the high accuracy and reliability of the variant detection but also the exclusion of potential systematic bias during sequencing. Further analysis revealed that C:G > T:A transitions were the most abundant, followed by T:A > C:G, while T:A > A:T transversions were the least frequent (Figure 1B).

SNP functional annotation results revealed the distribution patterns of variants across different genomic functional regions (Table 3). The vast majority of SNPs were enriched in non-coding regions: intronic regions contained an average of 1,821,812 SNPs (accounting for 56.4%), and intergenic regions contained an average of 1,223,203 SNPs (accounting for 37.9%). This aligns with the “Neutral Theory” of molecular evolution, which posits that non-coding regions are subject to less selection pressure and possess a greater capacity to tolerate variations. In gene regulation-related regions, upstream and downstream areas contained an average of 66,370 and 71,264 SNPs, respectively, suggesting they may participate in gene expression regulation by influencing transcription initiation or post-transcriptional modifications; meanwhile, the average number of SNPs in upstream/downstream overlapping regions was 10,517. In coding regions, synonymous mutations averaged 35,063; while not altering amino acid sequences, they may affect translation efficiency. Notably, non-synonymous mutations averaged 12,458. Although non-synonymous mutations account for a very small proportion, because they directly result in amino acid changes, they are often eliminated by strong purifying selection. Therefore, these retained non-synonymous mutations are often key potential factors driving phenotypic variation (such as growth rate and meat quality traits) and disease susceptibility, serving as important candidate loci for subsequent functional gene mining.

### 3.3. Demographic History and Genetic Status

**Figure 2:**
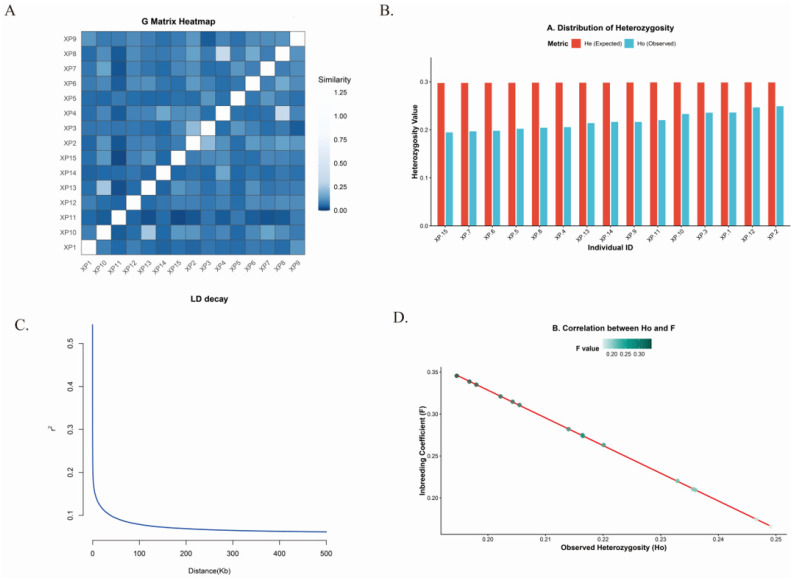
Genetic diversity and population structure analysis of the Xupu goose: (**A**) Genomic relationship matrix (G-matrix) heatmap. The color gradient represents the genomic kinship coefficient, with lighter blocks indicating higher pairwise relatedness, revealing family substructures within the population. (**B**) Distribution of heterozygosity. The bar chart compares the expected heterozygosity (*H_E_*, red) and observed heterozygosity (*H_O_*, blue) for each sample. (**C**) Linkage disequilibrium (LD) decay. (**D**) Correlation between observed heterozygosity (*H_O_*) and inbreeding coefficients (F). The scatter plot illustrates the linear regression between *H_O_* (x-axis) and F (y-axis), with point color intensity corresponding to the magnitude of the *F* value.

In this study, the average total length of ROH in the Xupu goose genome was 230.2 Mb, ranging from 195.1 to 272.9 Mb. Analysis of the segment size distribution (Figure 1E) revealed that short fragments (0.1–0.2 Mb) were the most abundant, totaling 7056 segments and accounting for 54.13% of the total, whereas long segments (>1 Mb) comprised only 1.76% (Supplementary Table S1). This dominance of short segments suggests the influence of ancient common ancestors or historical bottleneck effects rather than recent inbreeding—a conclusion further supported by the rapid decrease in frequency as ROH length increases. Consequently, the calculated average genomic inbreeding coefficient ($F_{ROH}$) was 0.204 (range: 0.173–0.241) (Figure 1D) (Supplementary Table S2). Collectively, these results demonstrate that while the Xupu goose population faces moderate inbreeding pressure. This moderate level of inbreeding is consistent with the breed’s history of closed-population conservation. Given the limitations of historical pedigree depth, the $F_{ROH}$ was calculated to provide a precise estimate of individual autozygosity and population inbreeding levels. Regarding heterozygosity and inbreeding levels (Figure 2B), the observed heterozygosity ($H_{O}$) ranged from 0.194 to 0.249 (mean 0.217), whereas the expected heterozygosity ($H_{E}$) remained stable, varying narrowly between 0.297 and 0.298 (mean 0.298). The average $H_{E}$ was significantly higher than the average $H_{O}$. Furthermore, linear regression analysis (Figure 2D) revealed a distinct negative correlation between $H_{O}$ and F. Collectively, these findings indicate a degree of heterozygote deficiency and inbreeding within the population.

The genomic relationship matrix (G-matrix) heatmap (Figure 2A) visualizes the kinship structure among the 15 sequenced individuals. Visual inspection of the kinship heatmap revealed a generally moderate background relatedness across the population, characterized by predominant dark blue coloration. However, distinct clusters of elevated genomic similarity (lighter hues) were observed among specific individuals. To further validate this observed substructure, a Principal Component Analysis (PCA) was conducted (Supplementary Figure S1). The PCA results strikingly corroborated the G-matrix patterns, with the first principal component (PC1) accounting for an exceptionally high 66.23% of the total genetic variance. Rather than forming a single panmictic cloud, the 15 individuals exhibited clear stratification along the PC1 axis, aggregating into corresponding sub-clusters.

The decay pattern of Linkage Disequilibrium (LD) reflects the population’s recombination history and serves as a critical determinant of the resolution of association mapping. Analysis of LD decay (Figure 2C) reveals that the linkage disequilibrium coefficient (*r*^2^) exhibits a rapid decline as physical distance increases. Notably, the curve drops precipitously within the short-range interval of 0–100 kb, after which it plateaus and stabilizes at a relatively low level.

### 3.4. Genome-Wide Detection of Selection Signatures Using iHS

**Figure 3:**
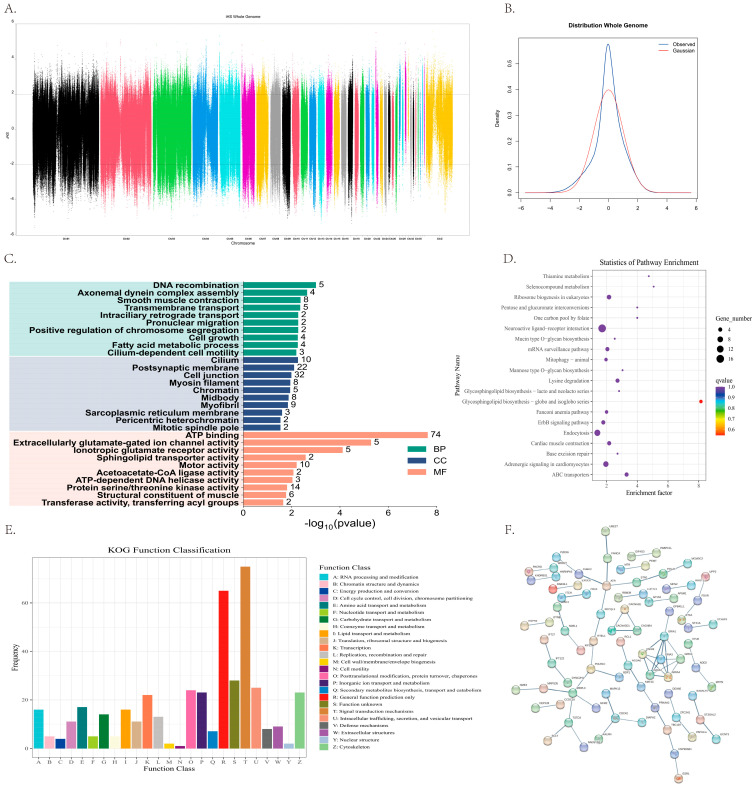
Comprehensive Analysis of Genomic Genetic Diversity and Positive Selection Signatures in Xupu Goose: (**A**) Manhattan plot of genome-wide iHS selection signals; (**B**) Genome-wide distribution of iHS statistics: comparison between the score distribution and the theoretical normal distribution; (**C**) Bar plot of GO functional enrichment; (**D**) Statistical plot of KEGG pathway enrichment: major metabolic pathways and biological systems associated with candidate genes within selected regions; (**E**) Statistical plot of KOG functional classification; (**F**) Statistical plot of KOG functional classification.

To investigate signatures of recent positive selection associated with the domestication of the Xupu goose, a genome-wide scan was performed using the Integrated Haplotype Score (iHS) method. First, the raw iHS values were standardized to facilitate comparison. The resulting genome-wide frequency distribution (Figure 3B) revealed that the bulk of the observed iHS values (blue curve) aligned closely with the theoretical standard normal distribution (red curve), exhibiting a typical bell-shaped pattern. This distributional pattern is critical for establishing statistical significance, as extreme values serve as indicators of potential targets of selection. Subsequently, a Manhattan plot (Figure 3A) was constructed to visualize the spatial distribution of these candidate selection signatures. Using a significance threshold of |iHS| > 2, multiple genomic regions enriched for selection signals were identified. Notably, distinct signal peaks were observed on autosomes 1, 2, and 4 (Chr1, Chr2, Chr4), and on the sex chromosome (ChrZ).

### 3.5. Gene Annotation and GO/KEGG Pathway Enrichment Analysis

To further elucidate the biological functions of these selected regions, the identified significant SNPs were mapped to the reference genome for gene annotation. A total of 3235 potential candidate genes were identified. Subsequently, functional enrichment analysis was performed on these genes using the Gene Ontology (GO) and KEGG databases.

GO functional enrichment analysis revealed that the identified candidate genes were significantly enriched across three main categories (Figure 3C): Biological Process (BP), Cellular Component (CC), and Molecular Function (MF). The top five most significantly enriched GO terms within each category are detailed (Table 4). In the BP category, the genes were predominantly involved in DNA recombination, smooth muscle contraction, and fatty acid metabolic process, along with other processes such as axonemal dynein complex assembly, transmembrane transport, and cell growth. Regarding the CC category, the candidate gene products were mainly localized in the cell junction, postsynaptic membrane, and cilium, while also showing significant enrichment in muscle-related components like myofibril and myosin filament. Within the MF category, ATP binding exhibited the highest level of significance and the largest gene count, followed by protein serine/threonine kinase activity and motor activity. Furthermore, enrichment in terms such as ionotropic glutamate receptor activity, sphingolipid transporter activity, and structural constituent of muscle. The Xupu goose population possesses specific genetic characteristics related to fat deposition, muscle development, and neural regulation. KEGG pathway enrichment analysis revealed (Figure 3D) that the candidate genes under selection were primarily involved in Glycosphingolipid biosynthesis, Neuroactive ligand-receptor interaction, Selenocompound metabolism, and ABC transporters. The top ten most significantly enriched pathways are listed in detail (Table 5).

KOG functional classification results revealed that the candidate genes were distributed across all 25 functional categories (Figure 3E). Among them, signal transduction mechanisms, General function prediction only, and Function unknown represented the three most enriched categories. Notably, the number of genes involved in Signal transduction mechanisms reached 75, indicating their predominant role in the identified selection signals. In addition, a significant number of genes were associated with Lipid transport and metabolism, Posttranslational modification, protein turnover, chaperones, and the cytoskeleton. These findings suggest that the evolutionary selection process in this population is highly concentrated on pathways related to cellular signaling, protein regulation, and structural development.

Protein–protein interaction network analysis revealed tight interactions among proteins encoded by the candidate genes (Figure 3F), forming multiple distinct functional modules. Within the network structure, the GRIA gene family exhibited high connectivity, constituting one of the core modules. This finding aligns perfectly with the significant GO term “ionotropic glutamate receptor activity”, suggesting that neural signaling and endocrine regulation play pivotal roles in defining the breed’s characteristics. Concurrently, the ATR protein emerged as another major hub node, interacting closely with FANCA and CHEK2, thereby forming a functional sub-network governing DNA repair and cell cycle surveillance.

## 4. Discussion

### 4.1. Genome-Wide Variation Patterns

Whole-genome resequencing has emerged as a pivotal tool for characterizing the genetic structure of livestock populations and evaluating germplasm resources [24]. In this study, we leveraged high-throughput WGS data to investigate the genetic diversity and signatures of selection within the Xupu goose population. It is important to note that the average sequencing depth of 7× may limit the detection of rare variants. However, stringent filtering criteria were applied to ensure that the common SNPs used for population structure and selection signature analyses are high-confidence variants. Post-quality control (QC) analysis revealed that the average GC content (~45%) and mapping rate (~98%) were consistent with those reported for other goose breeds [11,20], ensuring the reliability and accuracy of the data for subsequent analyses. Single-Nucleotide Polymorphisms (SNPs) serve as primary markers for assessing genetic diversity and are crucial for reconstructing evolutionary trajectories [25]. In the present study, statistical analysis identified an average of approximately 4.272 million SNPs per individual in the Xupu goose samples. The Ti/Tv ratio of 2.49 observed in this study falls well within the typical range reported for vertebrate genomes. This consistency not only attests to the high accuracy and reliability of the variant detection results, but also effectively rules out potential systematic biases introduced during the sequencing process. The genomic distribution of SNPs is consistent with the findings of Joanna Grzegorczyk et al. [26], being primarily concentrated in intergenic and intronic regions. Analysis of zygosity distribution reveals that the average number of heterozygous sites in Xupu goose samples generally exceeds that of homozygous sites; the observed proportion of heterozygous variants of 54% is within a reasonable range. Typically, long-term high-intensity artificial selection leads to a rapid increase in population homozygosity [27,28]; however, as a superior indigenous breed, the relatively high heterozygosity of the Xupu goose suggests that the population has not experienced a severe genetic bottleneck, thereby possessing potential for environmental adaptation and breeding plasticity. Although non-synonymous mutations account for only a minute proportion of variations, they are often subject to strong purifying selection and eliminated because such mutations can directly lead to amino acid alterations [29].

The length and frequency of ROH reflect individual relatedness; longer and more frequent ROH segments indicate a higher probability of consanguinity, with different segment lengths corresponding to ancient and recent inbreeding histories, respectively [30,31]. This study revealed that ROH in the Xupu goose genome were predominantly short segments (0.1–0.2 Mb), with their frequency decreasing rapidly as segment length increased. The extremely low proportion of long ROH segments (>1 Mb) was observed in the Xupu goose (accounting for only 1.76%). The findings suggest that the observed genomic homozygosity is likely attributable to ancient common ancestry or historical population bottlenecks [32].

Genetic diversity is the core for evaluating the value of livestock and poultry germplasm resources, with expected ($H_{E}$) and observed ($H_{O}$) heterozygosity serving as key indicators for measuring population variation levels [33,34]. Meanwhile, the genomic inbreeding coefficient ($F_{ROH}$) calculated based on runs of homozygosity (ROH) sensitively reflects the loss of population heterozygosity and the depletion trend of genetic variation [35]. For context, Huang et al. [36] reported an average $H_{O}$ of 0.261 and $F_{ROH}$ of 0.223 in the Shitou goose population. Similarly, Zhang et al. [37] observed an average $H_{O}$ of 0.345 alongside a notably elevated $F_{ROH}$ of 0.352 in the Landes breed. In contrast, the Xupu goose population in this study exhibited a lower average $H_{O}$ (0.217), while $H_{E}$ remained at a higher level (0.298), with an average $F_{ROH}$ of 0.204. The genomic inbreeding level of the Xupu goose is comparable to that of other conserved breeds [38], yet remains significantly lower than that observed in the intensively selected Landes breed. Notably, although an $F_{ROH}$ of 0.204 suggests moderate inbreeding pressure, its genomic landscape is characterized by the absolute predominance of short ROH segments and a low proportion of non-synonymous mutations ($P_{n}$ = 0.313). This unique combination of “high $F_{ROH}$, predominant short segments, and low $P_{n}$ indicates that the population’s homozygosity primarily stems from long-term historical genetic drift or ancient ancestral bottleneck effects, rather than recent high-intensity inbreeding. This finding aligns with the “managed balanced population” concept proposed by Cendron et al. [39], which maintains breed characteristics while effectively mitigating the risk of recent inbreeding depression. Furthermore, the gap between the high $H_{E}$ level and $H_{O}$ in Xupu geese reveals a unique “heterozygosity potential space”, indicating for future breeding improvement. As stated by Groeneveld et al. [40], indigenous breeds often experience a phased increase in inbreeding coefficients during conservation breeding, which not only elucidates the formation mechanism of the current moderate inbreeding level in Xupu geese but also provides a scientific basis for subsequent marker-assisted selection and sustainable utilization of germplasm resources.

To further elucidate the genetic architecture underlying these metrics, population structure was assessed using a genomic relationship matrix (G-matrix) and Principal Component Analysis (PCA). The G-matrix heatmap revealed specific clusters of elevated genomic similarity. This structural stratification was strongly corroborated by the PCA projection, where the first principal component (PC1) accounted for a substantial 66.23% of the total genetic variance, grouping individuals into distinct sub-clusters. This dual line of evidence confirms the presence of cryptic family-lineage substructures within the ex situ conservation flock. Complementing these findings, Linkage Disequilibrium (LD) decay analysis demonstrated a rapid decline in $r^{2}$ over short physical distances—a pattern highly concordant with the predominance of short ROH segments. This reinforces the conclusion that while distinct family substructures exist, the overarching inbreeding signature primarily reflects historical genetic drift rather than recent, intensive consanguineous mating.

### 4.2. Genomic Signatures of Selection and Biological Functions

In this study, GO and KEGG enrichment analyses of genomic regions under selection, filtered by specific thresholds, revealed that genes such as *GRIA1*, *GRIA4*, *LEPR* and *GABRA* were significantly enriched in the “Neuroactive ligand-receptor interaction” pathway. AMPA receptors are tetrameric ion channels formed by the combinatorial assembly of *GRIA1*, *GRIA2*, *GRIA3*, and *GRIA4* subunits; they function as the primary mediators of fast excitatory neurotransmission in the central nervous system (CNS), facilitating rapid synaptic signal transduction [41]. Furthermore, the trafficking and expression of AMPA receptors play pivotal roles in long-term potentiation (LTP) and long-term depression (LTD), mechanisms that are intrinsically linked to learning, memory, and environmental adaptability in animals [42]. Relevant experiments in chicks have demonstrated that the interaction between NMDA and AMPA receptors plays a significant role in the regulation of food intake [43]. The *GABRA* gene family encodes the primary inhibitory receptors for gamma-aminobutyric acid (GABA), which are ubiquitously expressed and play critical roles in the mammalian central nervous system (CNS) [44]; dysfunction or loss of *GABRA* significantly impairs neural development [45]. The leptin receptor belongs to the Class I cytokine receptor family and is primarily responsible for mediating the majority of the biological effects of leptin (LEP) in both the central nervous system and peripheral tissues [46]. Studies have demonstrated that specific haplotype blocks within the *LEPR* gene are significantly associated with body weight at 49 and 70 days of age, and feed intake in broiler chickens, thereby confirming its pivotal role in regulating growth and appetite in poultry [47,48]. These neural pathways likely facilitated the domestication process by modulating feeding behavior and neural regulation, thereby enabling the geese to better adapt to the environmental conditions of captivity.

Subsequently, during the identification of traits associated with fat deposition and muscle growth, the *ACSS2*, *ACSS3*, and *PECR* genes were found to be enriched in the fatty acid metabolism pathway. Existing studies have indicated that *ACSS2* is primarily responsible for the conversion of acetate into acetyl-CoA [49]. In mouse models, *ACSS2* deficiency has been shown to significantly attenuate body weight gain under high-fat diet conditions and ameliorate hepatic steatosis, confirming its role in optimizing systemic fat storage and utilization through the selective regulation of genes related to lipid metabolism [50]. Similar to *ACSS2*, *ACSS3* is a pivotal gene in lipid metabolism regulation, functioning primarily to drive propionate catabolism by converting it into propionyl-CoA [51]. Furthermore, studies on *PRKAA2* have observed that its expression is significantly upregulated in ducks as dietary energy levels increase, suggesting its potential involvement in fatty acid regulation, lipid biosynthesis, and transport processes [52]. Concurrently, other findings have demonstrated a significant correlation between this gene and meat quality traits, specifically muscle tenderness and pH value [53]. *CMYA5* and *MTPN*, which are involved in the myosin filament pathway and the cell growth pathway, are also significant genes influencing muscle growth. *MTPN* promotes protein synthesis and induces cellular hypertrophy by upregulating the expression of various cardiac marker genes in cardiomyocytes. Regarding skeletal muscle, Shohei Shiraishi et al. [54] found that *MTPN* significantly increases the content of structural proteins in murine muscle, while a study by Wang et al. [55] demonstrated that *MTPN* promotes porcine myocyte differentiation and myotube hypertrophy, results which are consistent with previous reports. Concurrently, *MTPN* has also been identified as a crucial candidate gene regulating skeletal muscle growth and development in beef cattle. Furthermore, in studies on pork quality, *CMYA5* is regarded as a key candidate gene, with research showing it is significantly correlated with drip loss and intramuscular fat content [56,57].

However, lacking a comparative control breed renders the associations of these single-population sweeps putative. Therefore, future research should prioritize expanding the sample size and conducting cross-breed comparative analyses, complemented by multi-omics approaches and functional validation to fully elucidate these complex molecular networks.

## 5. Conclusions

This study provides a foundational whole-genome resequencing dataset for the “protected indigenous breed” Xupu goose. Population genomics analysis indicates that while the breed retains moderate genetic potential, it currently faces significant historical inbreeding pressure. Crucially, dual evidence from the G-matrix and PCA reveals the presence of a cryptic family substructure within the ex situ conserved population. This structural stratification accurately accounts for the observed heterozygote deficiency and confirms that the population’s homozygosity stems from historical background drift and closed-herd management rather than recent extreme inbreeding. Through intra-population Integrated Haplotype Score (iHS) scanning, we identified multiple candidate genes associated with the breed’s unique phenotypic traits. Genes such as *ACSS2*, *ACSS3* and *PECR* were identified as candidates for lipid metabolism and fatty liver deposition. Meanwhile, *CMYA5*, *MTPN* and *LEPR* were found to be associated with muscular development. While these single-population associations require future cross-breed and multi-omics validation, these foundational genomic insights provide critical resources and theoretical guidance for optimizing conservation strategies, marker-assisted selection, and sustainable breeding programs for this indispensable waterfowl resource.

## Figures and Tables

**Table 1:** Statistical Summary of Sequencing Data Quality for the Xupu Goose.

Item	Raw Data	Raw Reads	Clean Data	Clean Reads	Q20	Q30	GC
XP.1	7.28	24,271,632	7.21	24,034,909	97.01	90.61	43.75
XP.2	7.87	26,227,449	7.78	25,934,237	97.86	93.14	44.52
XP.3	7.36	24,527,512	7.28	24,276,569	96.59	89.75	43.59
XP.4	6.71	22,364,543	6.64	22,145,940	96.76	89.98	43.61
XP.5	6.31	21,029,977	6.24	20,798,313	95.86	87.51	44.23
XP.6	5.96	19,863,366	5.90	19,662,647	97.73	92.70	43.81
XP.7	7.87	19,277,105	7.79	19,073,643	97.41	91.73	43.98
XP.8	7.84	21,761,892	7.76	21,536,171	97.66	92.50	43.77
XP.9	8.09	23,672,418	8.01	23,421,840	97.39	91.69	44.06
XP.10	6.42	26,225,799	6.36	25,957,962	97.16	91.27	43.65
XP.11	6.31	26,142,798	6.25	25,875,170	96.92	90.54	43.54
XP.12	5.60	26,981,587	5.54	26,711,713	97.62	92.35	43.68
XP.13	5.78	21,395,240	5.72	21,184,296	97.80	92.99	43.31
XP.14	6.53	21,048,807	6.46	20,842,462	97.26	91.34	43.43
XP.15	7.10	18,652,074	7.03	18,464,475	97.38	91.66	43.62
Average	6.87	22,896,147	6.80	22,661,356	97.23	91.32	43.77

Note: Raw Data, total output of raw sequencing data (Gb); Raw Reads, total count of raw reads; Clean Data, high-quality data after filtering (Gb); Clean Reads, total count of clean reads; Q20 and Q30, percentage of bases with Phred quality scores > 20 (99% accuracy) and > 30 (99.9% accuracy), respectively; GC, proportion of Guanine and Cytosine bases.

**Table 2:** Statistics of SNP variations identified across Xupu goose samples.

Item	SNP	Ti	Tv	Ti/Tv (%)	He	Ho
XP.1	4,489,191	3,199,920	1,289,271	2.48	2,520,626	1,968,565
XP.2	4,530,104	3,236,055	1,294,049	2.50	2,658,006	1,872,098
XP.3	4,449,327	3,171,516	1,277,811	2.48	2,526,259	1,923,068
XP.4	4,226,956	3,016,067	1,210,889	2.49	2,188,910	2,038,046
XP.5	4,022,685	2,874,688	1,147,997	2.50	2,106,188	1,916,497
XP.6	4,026,556	2,876,173	1,150,383	2.50	2,065,952	1,960,604
XP.7	3,971,316	2,835,429	1,135,887	2.50	2,036,545	1,934,771
XP.8	4,141,730	2,956,349	1,185,381	2.49	2,155,020	1,986,710
XP.9	4,141,730	3,091,042	1,239,409	2.49	2,305,167	2,025,284
XP.10	4,516,093	3,217,257	1,298,836	2.48	2,520,962	1,995,131
XP.11	4,445,890	3,165,613	1,280,277	2.47	2,366,219	2,079,671
XP.12	4,445,890	3,298,795	1,330,674	2.48	2,661,174	1,968,295
XP.13	4,224,263	3,011,218	1,213,045	2.48	2,273,180	1,951,083
XP.14	4,198,350	2,993,075	1,205,275	2.48	2,290,077	1,908,273
XP.15	3,877,534	2,766,182	1,111,352	2.49	2,008,960	1,868,574
Average	4,271,994	3,047,292	1,224,702	2.49	2,312,216	1,959,778

Note: SNP, the total number of single-nucleotide polymorphism sites detected within the sample; Ti (Transition), refers to the substitution between bases of the same chemical type; Tv (Transversion), refers to the substitution between bases of different chemical types; Ti/Tv, the ratio of the frequency of transitions to the frequency of transversions; *H_e_*, Expected Heterozygosity; *H_o_*, Observed Heterozygosity.

**Table 3:** Statistics of SNP functional annotation.

Annotation Type	Mean ± SD
**Upstream**	66,369.93 ± 3090.43
**Alternative Splicing**	143.53 ± 14.30
**Synonymous**	35,062.87 ± 1438.87
**Non-synonymous**	12,458.07 ± 747.76
**Intron**	1,821,812.33 ± 73,183.17
**Downstream**	71,263.67 ± 3329.00
**Up/Downstream**	10,775.33 ± 426.38
**Intergenic**	1,223,202.67 ± 52,416.49

**Table 4:** Top significantly enriched GO terms of candidate genes in Xupu goose.

Item	ID	Terms	N	*p*	Genes Name
BP	GO:0006310	DNA recombination	5	9.86 × 10^−4^	*ACTR8*/*RECQL5*/*MND1*/*RTEL1*/*LIG3*
	GO:0070286	Axonemal dynein complex assembly	4	2.28 × 10^−3^	*ZC3H7A*/*CCDC62*/*WDR86*/*WDR88*
	GO:0006939	Smooth muscle contraction	8	4.26 × 10^−3^	*SYCP1*/*MAD1L1*/*ERC2*/*HTR1D*/*LOC106039360*/*GOLGA3*/*ERC1*/*CCDC73*
	GO:0055085	Transmembrane transport	5	4.41 × 10^−3^	*ABCC10*/*LOC106041486*/*FLVCR2*/*ABCC5*/*SPNS*
	GO:0006631	Fatty acid metabolic process	4	5.54 × 10^−3^	*PECR*/*ACSS3*/*AASDH*/*ACSS2*
CC	GO:0005929	Cilium	10	5.44 × 10^−3^	*ANKS6*/*ARL13B*/*WDR86*/*NEK1*/*IFT122*/*RAB12*/*WDR88*/*BBS12*/*NEK10*/*CEP131*
	GO:0030054	Cell junction	32	1.02 × 10^−2^	*GPHN*/*LOC106046349*/*DTNB*/*GRIA3*/*GABRA4*/*GRIA2*/*GRIA1*/*SRGAP2*/*SYNE2*/*GRIK4*/*GRIA4*, etc.
	GO:0032982	Myosin filament	8	1.13 × 10^−2^	*ERC1*/*GOLGA3*/*CCDC73*/*IQCE*/*CMYA5*/*MAD1L1*/*SYCP1*/*ERC2*
	GO:0030016	Myofibril	9	1.42 × 10^−2^	*ERC1*/*GOLGA3*/*IQCE*/*CCDC73*/*CMYA5*/*SYCP1*/*MAD1L1*/*ERC2*/*BDP1*
	GO:0033017	Sarcoplasmic reticulum membrane	3	2.48 × 10^−2^	*ATP2A2*/*FKBP1B*/*FKBP6*
MF	GO:0005524	ATP binding	2	2.34 × 10^−8^	*KASH5*
	GO:0005234	Extracellular glutamate-gated ion channel activity	74	5.16 × 10^−6^	*UBE2T*/*MAP3K15*/*CSNK1G3*/*PIK3CD*/*ABCB7*/*LOC106043431*/*DPH6*/*AASDH*/*HSP90AB1*, etc.
	GO:0004970	Ionotropic glutamate receptor activity	5	7.71 × 10^−5^	*GRIA3*/*GRIA4*/*GRIA1*/*GRIK4*/*GRIA2*
	GO:0046624	Sphingolipid transporter activity)	14	2.22 × 10^−2^	*MAP3K15*/*CSNK1G3*/*KALRN*/*CAMKK2*/*STK24*/*ATR*/*PRKAA2*/*NEK1*/*BRSK2*/*TTBK2*/*NEK10*/*ADCK1*/*CPNE3*/*MAP3K19*
	GO:0003774	Motor activity	10	6.18 × 10^−3^	*ERC1*/*GOLGA3*/*CCDC73*/*MAD1L1*/*SYCP1*/*ERC2*

Note: GO Category refers to the primary domains of the Gene Ontology (GO) system. It comprises three fundamental classifications: BP (Biological Process), which describes the broad biological objectives or biochemical pathways to which a gene product contributes; CC (Cellular Component), which specifies the precise locations within the cell where a gene product exerts its function; and MF (Molecular Function), defines the fundamental activities of a gene product at the molecular level. Terms provides the specific functional descriptions associated with each GO entry; N indicates the number of candidate genes from this study that are significantly enriched within a specific GO term. Genes Name: Lists the specific symbols of the candidate genes associated with the corresponding GO term; *p* (*p*-value), the statistical significance of the enrichment analysis; a value of *p* < 0.05 typically indicates that the enrichment of candidate genes in that pathway is highly significant and unlikely to have occurred by chance; Genes Name, lists the specific symbols of the candidate genes associated with the corresponding GO term.

**Table 5:** KEGG pathway enrichment analysis of candidate genes in Xupu goose.

Pathway	Gene Ratio	*p*	N	Genes Name
Glycosphingolipid biosynthesis—globo and isoglobo series	2.15	5.25 × 10^−3^	3	*B3GALT5*/*FUT9*/*ST3GAL2*
Neuroactive ligand-receptor interaction	11.1	2.33 × 10^−2^	16	*CNR1*/*GABRA4*/*GRIA1*/*GRIA2*/*GRIA3*/*GRIA4*/*GRIK4*/*HTR1D*/*LEPR*/*LOC106032793*/*LOC106039360*/*LOC106045569*/*LPAR4*/*P2RX6*/*P2RY8*/*SSTR2*
ABC transporters	2.87	3.19 × 10^−2^	4	*ABCB7*/*ABCC10*/*ABCC5*
Selenocompound metabolism	1.45	5.77 × 10^−2^	2	*MTR*/*SCLY*
Lysine degradation	2.87	5.89 × 10^−2^	4	*KMT2C*/*LOC106036705*/*NSD2*/*PRDM2*
Thiamine metabolism	1.43	6.49 × 10^−2^	2	*AK7*/*LOC106046413*
Adrenergic signaling in cardiomyocytes	5.03	6.64 × 10^−2^	7	*ATP2A2*/*CACNA2D1*/*CACNB4*/*LOC106048956*/*MAPK12*/*PLCB2*/*RAPGEF4*
Ribosome biogenesis in eukaryotes	3.59	8.13 × 10^−2^	5	*LOC125184964*/*LOC125184965*/*RCL1*/*SPATA5*/*XRN1*
Pentose and glucuronate interconversions	1.44	8.78 × 10^−3^	2	*CRPPA*/*XYLB*
One carbon pool by folate	1.42	8.78 × 10^−3^	2	*LOC106043431*/*MTR*

Note: Pathway, Refers to the specific name assigned to a metabolic or signaling pathway; Gene Ratio, The ratio of the number of candidate genes enriched in a specific pathway to the total number of candidate genes annotated in the entire dataset; *p* (*p*-value), The statistical significance of the enrichment analysis; a value of *p* < 0.05 typically indicates that the enrichment of candidate genes in that pathway is highly significant and unlikely to have occurred by chance; N, Indicates the number of candidate genes from the dataset that are significantly enriched within a particular pathway; Genes Name, Lists the symbols of candidate genes associated with the specific pathway.

## Data Availability

All data generated or analyzed during this study are included in this published paper.

## Supplementary Materials

The following supporting information can be downloaded at https://www.mdpi.com/article/10.3390/biology15060479/s1. Figure S1: PCA Analysis of XP Population; Table S1: Statistics of ROH Length Distribution; Table S2: Statistics of Individual Inbreeding Coefficients (Froh).
