# Supplementary material for: Genomic Evaluation of the Genetic Structure and Analysis of Selective Evolutionary Signatures of Xupu Goose
Source: Biology (Basel). 2026 Mar 17;15(6):479. doi: 10.3390/biology15060479 (PMC13023516; doi:10.3390/biology15060479)
Supplement: Supplementary file 1 [file biology-15-00479-s001.zip › Supplementary.pdf]

Supplementary Table S1. Statistics of ROH Length Distribution.

ROH Length Class (Mb)	Count	Percentage
**0.1-0.2**	**7,056**	**54.13%**
**0.2-0.3**	**2,583**	**19.81%**
**0.3-0.4**	**1,301**	**9.98%**
**0.4-0.5**	**778**	**5.97%**
**0.5-0.6**	**447**	**3.43%**
**0.6-0.7**	**264**	**2.03%**
**0.7-0.8**	**173**	**1.33%**
**0.8-0.9**	**122**	**0.94%**
**0.9-1**	**82**	**0.63%**
**>1**	**230**	**1.76%**

Supplementary Table S2. Statistics of Individual Inbreeding Coefficients (Froh)

Item	Total ROH Length (Mb)	FROH
**XP.3**	**195.1**	**0.1726**
**XP.14**	**195.6**	**0.1731**
**XP.15**	**196.0**	**0.1734**
**XP.1**	**202.4**	**0.1790**
**XP.2**	**210.5**	**0.1863**
**XP.5**	**219.4**	**0.1941**
**XP.13**	**225.2**	**0.1992**
**XP.7**	**227.7**	**0.2015**
**XP.10**	**230.1**	**0.2036**
**XP.6**	**240.2**	**0.2125**
**XP.4**	**263.9**	**0.2335**
**XP.11**	**269.8**	**0.2387**
**XP.8**	**270.7**	**0.2395**
**XP.9**	**272.9**	**0.2414**
**XP.12**	**232.8**	**0.2040**
**Average**	**230.2**	**0.2040**
