# Supplementary figures and images for: Genomic Evaluation of the Genetic Structure and Analysis of Selective Evolutionary Signatures of Xupu Goose
Source: Biology (Basel). 2026 Mar 17;15(6):479. doi: 10.3390/biology15060479 (PMC13023516; doi:10.3390/biology15060479)

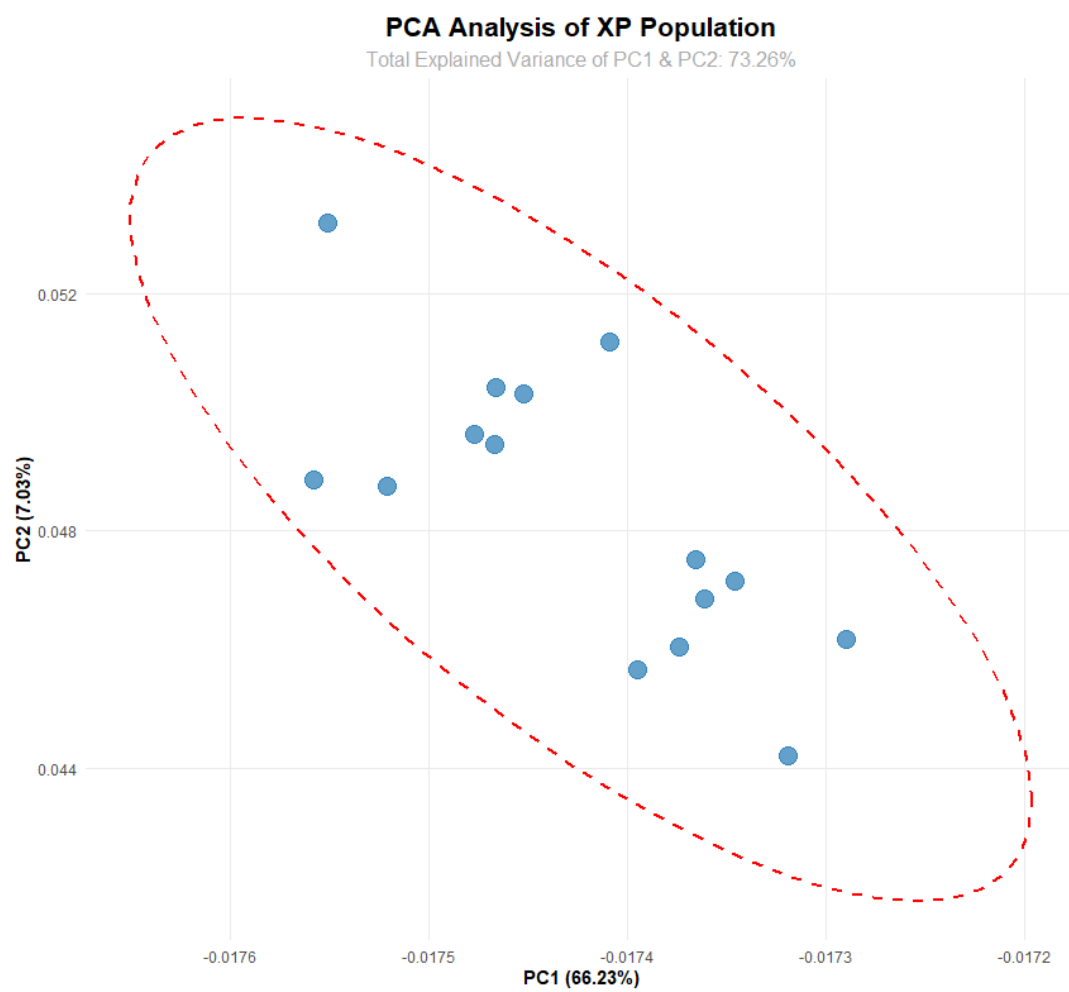

**Supplementary Figure S1. PCA Analysis of XP Population**

Supplement: Supplementary file 1 [file biology-15-00479-s001.zip › biology-4181491-supplementary.pdf]
